# Supplementary material for: Distribution, diversity and persistence of Listeria monocytogenes in swine slaughterhouses and their association with food and human listeriosis strains
Source: PLoS One. 2020 Aug 6;15(8):e0236807. doi: 10.1371/journal.pone.0236807 (PMC7410256; doi:10.1371/journal.pone.0236807)

S1 Fig. Pulsotype diversity of L. *monocytogenes* isolated from four swine slaughterhouses in Quebec, Canada

A. Pulsotype diversity according to the seasons (A:Fall, B:Winter, C:Spring, D:Summer)

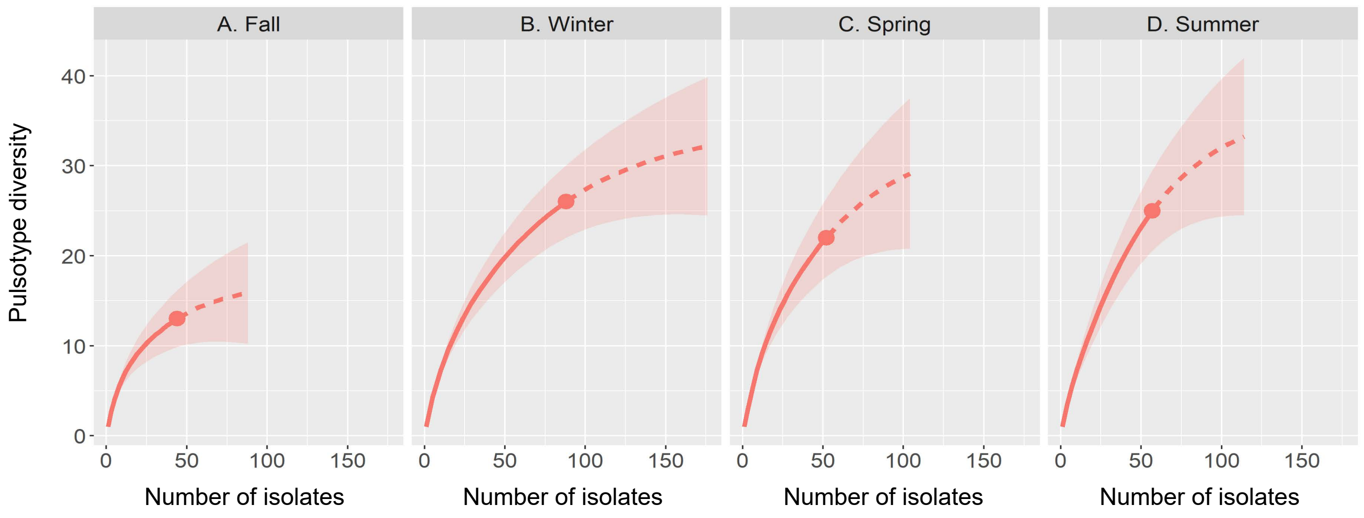

B. Pulsotype diversity according to slaughterhouses (A, B, C, D)

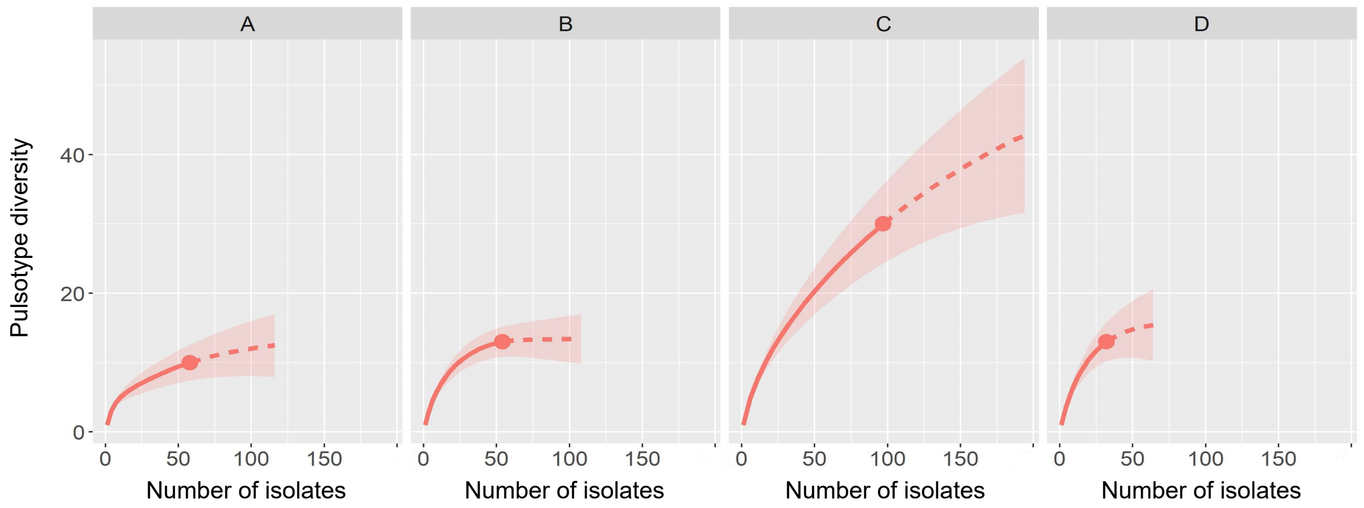

C. Pulsotype diversity according to the operation areas in the slaughterhouse (A:Lairage, B:Slaughtering and bleeding, C:Dehairing and evisceration, D:Chilling and hanging, E:Cutting and deboning)

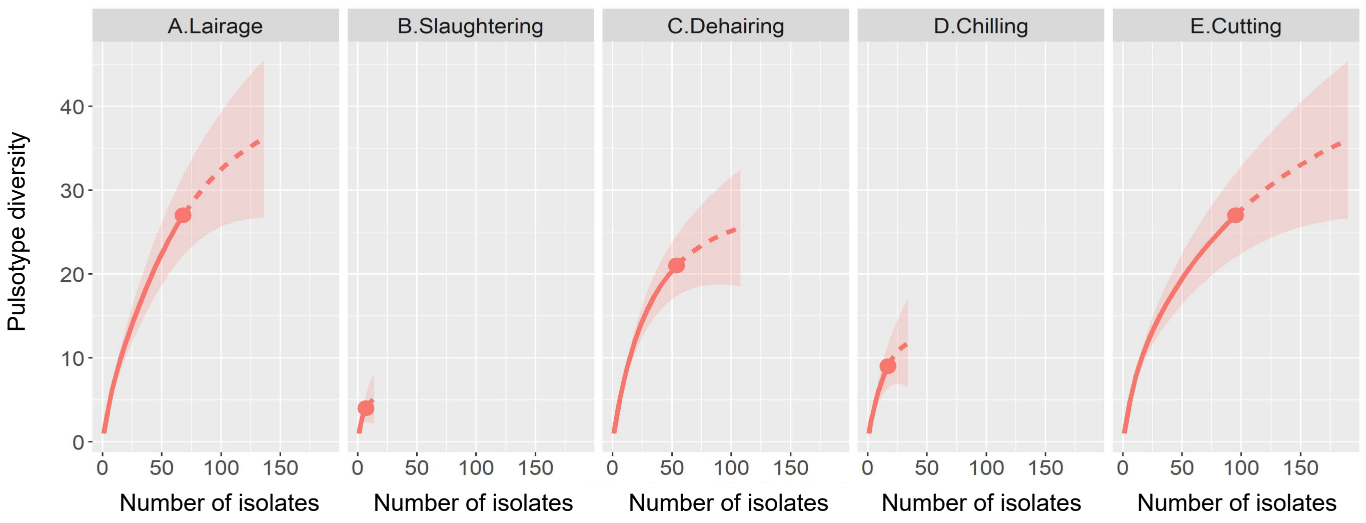

Supplement: S1 Fig — A. Pulsotype diversity according to the seasons (A: Fall, B: Winter, C: Spring, D: Summer). B. Pulsotype diversity according to slaughterhouses (A, B, C, D). C. Pulsotype diversity according to the operation areas in the slaughterhouse (A: Lairage, B: Slaughtering and bleeding, C: Dehairing and evisceration, D: Chilling and hanging, E: Cutting and deboning) (PDF) [file pone.0236807.s001.pdf]
