# Supplementary material for: Distribution, diversity and persistence of Listeria monocytogenes in swine slaughterhouses and their association with food and human listeriosis strains
Source: PLoS One. 2020 Aug 6;15(8):e0236807. doi: 10.1371/journal.pone.0236807 (PMC7410256; doi:10.1371/journal.pone.0236807)
Supplement: S1 Table — (PDF) [file pone.0236807.s002.pdf]

S1 Table. Sampling of four slaughterhouses in Quebec, Canada, for the isolation of *Listeria monocytogenes*.

Operation areas and specific sites, machines and materials	Number of samples per visit, per slaughterhouse[a]	In regular contact with animal carcasses and meat	Category[b]	Subjected to in-site cleaning and sanitation (with QAC)
**Lairage**				
Concrete slab of the Main entrance to the slaughterhouse (5 surfaces X 1 m^2 each, with the same lab wipe)	1	No	Environment	NA
Pen for sick animals (1 pen; 5 samples; 1 m^2 each)	5	No	Environment	NA
Pens for normal animals (9 pens; 5 samples per pen; 1 m^2 each)	45	No	Environment	NA
Hallway floor (5 surfaces X 1 m^2 each, with the same lab wipe)	1	No	Environment	NA
Hallway wall (5 surfaces X 1 m^2 each, with the same lab wipe)	1	No	Environment	NA
**Slaughtering and bleeding and dehairing**				
Floor (1 m^2)	6	No	Environment	NA
Wall (3 surfaces X 1 m^2 each, with the same lab wipe)	2	No	Environment	NA
Ceiling (3 surfaces X 1 m^2 each, with the same lab wipe)	1	No	Environment	NA
Bleeding equipment (knife and sharpener)	1	Yes	Material	NA
Tables (3 tables X 1 m^2 each with the same lab wipe)	3	Yes	Material	NA
Scalding tank (5 surfaces X 1 m^2 each with the same lab wipe)	1	Yes	Machine	NA

Operation areas and specific sites, machines and materials	Number of samples per visit, per slaughterhouse[a]	In regular contact with animal carcasses and meat	Category[b]	Subjected to in-site cleaning and sanitation (with QAC)
Dehairing machine (5 surfaces X 1 m^2 each with the same lab wipe)	2	Yes	Machine	NA
Brushing machine (5 surfaces X 1 m^2 each with the same lab wipe)	2	Yes	Machine	NA
**Evisceration**				
Floor (1 m^2)	6	No	Environment	High
Wall (3 surfaces 1 m^2 each, with the same lab wipe)	2	No	Environment	High
Ceiling (3 surfaces, 1 m^2 each, with the same lab wipe)	1	No	Environment	Low
Clipping machine	1	SC	Machine	Low
Sternal splitter	1	SC	Machine	Low
Visceral container	3	SC	Material	Low
Back splitter	1	SC	Machine	Low
Degreasing knife	1	SC	Machine	Low
Knives	1	SC	Material	Low
Gloves	1	SC	Material	Low
Sharpener	1	SC	Material	Low
Apron	1	SC	Material	Low
Hooks (10 hooks with the same lab wipe)	3	SC	Material	Low
**Chilling and hanging**				
Door (1 m^2)	2	No	Environment	High
Floor (1 m^2)	3	No	Environment	High
Wall (1 m^2)	3	No	Environment	High
**Cutting and deboning**				
Floor (1 m^2)	5	No	Environment	High

Operation areas and specific sites, machines and materials	Number of samples per visit, per slaughterhouse[a]	In regular contact with animal carcasses and meat	Category[b]	Subjected to in-site cleaning and sanitation (with QAC)
Wall (3 surfaces X 1 m^2 each, with the same lab wipe)	2	No	Environment	High
Ceiling (3 surfaces X 1m^2 each, with the same lab wipe)	1	No	Environment	Low
Post	2	No	Environment	High
Fan	2	No	Environment	Low
Door (1 m^2)	2	No	Environment	High
Conveyor belt 1 (top and bottom sides)	6	SC	Environment	High
Conveyor belt 2 (top and bottom sides)	6	SC	Environment	High
Conveyor belt 3 (top and bottom sides)	6	SC	Environment	High
Conveyor belt 4 (top and bottom sides)	6	SC	Environment	High
Conveyor belt 5 (top and bottom sides)	6	SC	Environment	High
Saws	1	SC	Machine	Low
Knives (3 knifes per sample; total 9 knifes: 3 samples)	3	SC	Material	Low
Sharpeners (3 sharpeners per sample; total 9 sharpeners: 3 samples)	3	SC	Material	Low
Shelves (3 surfaces X 1m^2 each, with the same lab wipe)	3	SC	Material	Low

QAC: Quaternary ammonium compounds

NA: Not applicable: QAC were not used in these operation areas

High: surfaces which are more exposed to QAC

Low: surfaces which are weakly exposed to QAC

[a] Number of samples collected during one visit, per slaughterhouse. Each slaughterhouse was visited four times.

[b] Specific sampled sites, machines and materials were grouped into three categories (environment, material or machine) for statistical analyses.
